# Bidirectional Association Between Sleep and Brain Atrophy in Aging

**DOI:** 10.3389/fnagi.2021.726662

**Published:** 2021-12-08

**Authors:** Viktória Kokošová, Pavel Filip, David Kec, Marek Baláž

**Affiliations:** ^1^Department of Neurology, Faculty of Medicine, University Hospital Brno and Masaryk University, Brno, Czechia; ^2^Department of Neurology, First Faculty of Medicine, General University Hospital Prague and Charles University, Prague, Czechia; ^3^Center for Magnetic Resonance Research (CMRR), University of Minnesota, Minneapolis, MN, United States; ^4^First Department of Neurology, Faculty of Medicine, University Hospital of St. Anne and Masaryk University, Brno, Czechia

**Keywords:** brain aging, sleep, neuroimaging, structural brain integrity, functional brain integrity

## Abstract

Human brain aging is characterized by the gradual deterioration of its function and structure, affected by the interplay of a multitude of causal factors. The sleep, a periodically repeating state of reversible unconsciousness characterized by distinct electrical brain activity, is crucial for maintaining brain homeostasis. Indeed, insufficient sleep was associated with accelerated brain atrophy and impaired brain functional connectivity. Concurrently, alteration of sleep-related transient electrical events in senescence was correlated with structural and functional deterioration of brain regions responsible for their generation, implying the interconnectedness of sleep and brain structure. This review discusses currently available data on the link between human brain aging and sleep derived from various neuroimaging and neurophysiological methods. We advocate the notion of a mutual relationship between the sleep structure and age-related alterations of functional and structural brain integrity, pointing out the position of high-quality sleep as a potent preventive factor of early brain aging and neurodegeneration. However, further studies are needed to reveal the causality of the relationship between sleep and brain aging.

## Introduction

Aging is accompanied by a gradual loss of function and structural degeneration in all organ systems (López-Otín et al., 2013). In the central nervous system, the declining metabolic rate together with diminished effectiveness of the antioxidant system (Emir et al., 2011; Goyal et al., 2017) is reflected in impaired clearance and accumulation of metabolic waste products. Accumulated toxic waste products, e.g., amyloid beta, bind to microglial toll-like receptors and activate the complement system leading to phenotypic changes in the microglia (Walter et al., 2007; Wyss-Coray and Rogers, 2012). These microglial cells predominantly polarize into classically activated M1 state and increase the secretion of pro-inflammatory cytokines—interleukin-6, interleukin-1β, and tumor necrosis factor α (Wyss-Coray and Rogers, 2012; Cherry et al., 2014; Gomez-Nicola and Boche, 2015). Subsequent chronic inflammation contributes to macroscopic gray and white matter (GM and WM, respectively) atrophy (Perry and Teeling, 2013; Moreno-García et al., 2018). These changes in structure and function are considered to be the underlying basis of eventual cognitive decline (Suzuki et al., 2019), sleep disturbances (Cheng et al., 2013; Dubé et al., 2015; Carvalho et al., 2017), and may even lead to the development of neurodegenerative disorders (Akiyama et al., 2000; Salat et al., 2009; Power et al., 2019). The resultant overall performance decline represents a burden for the individual and for society.

Therefore, understanding the processes underlying brain aging is crucial to preventing the development of age-related diseases, and potentially also neurodegenerative disorders, opening a window for eventually targeted prevention and early treatment—the holy grail of a vast segment of neuroscientific research (Dai et al., 2018; Babazadeh et al., 2020; Soo et al., 2020). Particularly nutrition (Kelsey et al., 2010; Domenico and Giudetti, 2017), physical activity together with mental wellbeing (Steffener et al., 2016), and high-quality sleep (Cheng et al., 2013; Dubé et al., 2015; Carvalho et al., 2017) are, when appropriately managed, important modifiable protective factors against early brain aging.

The sleep of insufficient quality and length does not only immediately reduce the elimination of toxic waste products (Spira et al., 2013; Shokri-Kojori et al., 2018) and next-day cognitive performance (Scullin and Bliwise, 2015; Varga et al., 2016; Fogel et al., 2017), but is, in long run, also associated with advanced brain atrophy (Spira et al., 2016; Baillet et al., 2017; Sexton et al., 2017; Tahmasian et al., 2020) and a higher risk of developing dementia (Bah et al., 2019). The risk of the development of the most common type of dementia—Alzheimer’s disease—has been linked with sleep deprivation [as reviewed in Ju et al. (2014); Wu et al. (2019); Bishir et al. (2020); Wang and Holtzman (2020)]. Further, sleep disorders are associated with advanced brain aging and the development of neurodegenerative disorders (Kumar et al., 2012; Rupprecht et al., 2013; Chen et al., 2015; Li et al., 2016; Weihs et al., 2021). Regarding the essential role of sleep in brain homeostasis, we consider it to be one of the most important modifiable risk factors of brain aging.

Filling the void in the currently available literature, this review focuses on the link between brain aging and sleep—one of the key modifiable risk factors—through the viewing glass of various neuroimaging and neurophysiological methods.

## Neuroimaging Correlates of Brain Aging

There is wide agreement among neuroimaging studies that cortical and subcortical GM volume decreases with age (Resnick et al., 2003; Du et al., 2004; Raz et al., 2004; Allen et al., 2005; Walhovd et al., 2005; Mueller and Weiner, 2009; Eavani et al., 2018), with substantial inter-regional variance. The midline frontal cortex and structures underlying the default mode network (DMN) alongside the medial temporal lobe subsystem appear to be particularly susceptible to age-related changes (Sowell et al., 2003; Raz et al., 2004; Fjell et al., 2009). Accordingly, DMN regions are among the earliest to show abnormal amyloid deposition (Mintun et al., 2006). Akin to GM, cerebral WM exhibits regionally variable deterioration in aging (Walhovd et al., 2005; Sexton et al., 2014b; Liu et al., 2017). The first and most affected are frontal and temporal WM pathways (Salat et al., 2005; Sexton et al., 2014b; Liu et al., 2017). In contrast to GM, the degeneration pattern of WM has a shape of inverted “U,” i.e., the WM volume increases until the 50–60 s, and begins to decline afterward (Allen et al., 2005). Diffusion tensor imaging (DTI) sensitive to microstructural tissue properties (Song et al., 2005, 2003; Budde et al., 2007) provides information complementary to volumetry. Fractional anisotropy (FA) indicates that the uniformity of water diffusivity increases sharply in adolescence and early adulthood. Approximately at the age of 30, FA reaches its maximum, subsequently enters the plateau phase, with a consequent FA decline and radial diffusivity (RD) and mean diffusivity (MD) increase. Interestingly, the FA and RD peak happens almost 20 years earlier than macrostructural WM atrophy can be detected in structural images (Westlye et al., 2010). Thus, DTI represents a method capable of detecting microstructural changes preceding the development of irreversible macroscopic changes.

In addition to trophic changes, brain functional connectivity changes with age. In conformity with morphological tissue loss, the effects of aging were most consistent in highly overlapping regions of DMN and medial temporal lobe subsystem (Dennis and Thompson, 2014; Liu et al., 2018). The functional connectivity in these regions was significantly lower in older compared to young adults (Liu et al., 2018). Moreover, the decrease in functional connectivity was associated with amyloid deposition in DMN (Bero et al., 2012), which is predominantly deposited in highly metabolically active regions and may disrupt functional connectivity even before it causes any detectable atrophy (Sheline and Raichle, 2013).

## Neuroimaging Parameters Related to Sleep

The sleep of insufficient length and quality is associated with accelerated brain atrophy (Sexton et al., 2014a; Spira et al., 2016; Tahmasian et al., 2020). Concerning sleep duration, The National Sleep Foundation recommends optimally at least 7 h of sleep per day for adults, with respect to inter-individual variabilities in the sleep need (Hirshkowitz et al., 2015). Participants self-reporting sleep shorter than 7 h a day exhibited a significant longitudinal decrease of cortical thickness in fronto-temporal regions (Spira et al., 2016; Tahmasian et al., 2020). However, not only short but also sleep longer than 7 h was associated with higher cortical thinning rate, namely, in the left superior and middle frontal gyrus (Spira et al., 2016). Likewise, poor sleep quality was associated with decreased cortical thickness and higher cortical thinning rate in fronto-temporo-parietal regions, such as the medial superior frontal cortex and cingulate (Sexton et al., 2014a). Alike age-related GM atrophy, poor sleep seems to mostly affect fronto-temporal brain regions.

The integrity of WM tracts is also affected by sleep (Yaffe et al., 2016; Baillet et al., 2017; Sexton et al., 2017). Even though total WM volume was not associated with night-time sleep duration or self-evaluated sleep quality in either young or older adults (Y.-R. Lo et al., 2014; Liu et al., 2018; Aribisala et al., 2020), WM integrity impairment in sleep-deprived subjects was detected with the use of DTI. Specifically, short and fragmented sleep was linked with a diminution of WM integrity predominantly in fronto-temporal and fronto-subcortical WM tracts in middle-aged and older adults, reflected in decreased FA and increased MD and RD (Yaffe et al., 2016; Baillet et al., 2017; Sexton et al., 2017). Sleep appears to affect rather WM integrity with no significant correlation with macroscopic WM atrophy.

Furthermore, the functional connectivity of neuronal networks changes during the sleep-wake cycle (Horovitz et al., 2009; Sämann et al., 2010; Regen et al., 2016). Sleep deprivation was correlated with decreased resting-state functional connectivity in DMN (Sämann et al., 2010). Correspondingly, reduced DMN connectivity is favorable for restful sleep, and increased DMN connectivity is correlated with poor sleep efficiency and prolonged sleep-onset latency (Horovitz et al., 2009; Regen et al., 2016). Changes in functional connectivity may therefore participate in the development of sleep disorders.

## Neuroimaging and Neurophysiological Correlates of the Association Between Sleep and Brain Aging

As previously mentioned, sleep has an ample impact on the structural and functional changes of the brain during the life course—both sleep duration and quality are crucial (Sämann et al., 2010; Sexton et al., 2014a,^2017^; Spira et al., 2016; Yaffe et al., 2016; Baillet et al., 2017; Tahmasian et al., 2020).

Two types of sleep are distinguished –rapid-eye movement (REM) and non-rapid-eye movement sleep (NREM) sleep. NREM is further divided into three stages (N1, N2, and N3), based on distinct electroencephalographic features (Berry et al., 2017). These stages cycle during sleep and are highly complementary and in the long-run equally important for brain homeostasis (Vyazovskiy and Delogu, 2014).

Similar to brain morphology and function, sleep structure is also substantially impaired in senescence. Total sleep time, and proportionally mainly N3 sleep, becomes progressively shorter and more fragmented with subsequent diminution of sleep efficiency and quality (Dubé et al., 2015; Varga et al., 2016). At the level of sleep microarchitecture, the diminution of oscillatory neuronal activity, predominantly of sleep spindles (SS), and slow waves (SWs) is a characteristic of aging (Massimini et al., 2004; Murphy et al., 2009; Martin et al., 2013). Namely, the changes in SS amplitude and density dominate over frontal regions, while their duration is decreased predominantly over posterior electroencephalographic (EEG) derivations (Martin et al., 2013; Mander et al., 2017). On the other hand, the diminution of SW activity, density, slope, and amplitude occurs globally, with dominant affection over frontal and prefrontal regions (Mander et al., 2013; Dubé et al., 2015; Varga et al., 2016). In addition to differences in aging pattern of SS and SW, their formation and propagation retain high intraindividual stability in contrast to substantial interindividual variability (Massimini et al., 2004; De Gennaro et al., 2005; Rusterholz and Achermann, 2011), limiting the yield of simple cross-sectional studies. Nonetheless, EEG feature variability and their age-related changes bear a substantial association with age-related changes of GM and WM involved in their generation and subsequent spread (Buchmann et al., 2011; Piantoni et al., 2013; Saletin et al., 2013).

The neuronal activity of the thalamocortical loop plays a key role in SS generation during sleep (Berry et al., 2017; Fernandez and Lüthi, 2020). Correspondingly, the age-related fronto-temporal WM atrophy was, specifically in the tracts connecting thalamus to cortical areas and in corpus callosum and association fibers, correlated with the decrease of SS amplitude and density over frontal areas (Piantoni et al., 2013; Mander et al., 2017; Gaudreault et al., 2018). Furthermore, in concordance with SS shortening over posterior EEG derivations in aging (Mander et al., 2017), a higher GM volume in the cerebellum, hippocampus, cingulate, and parietal cortices was predictive of longer SS duration in both young and older adults (Fogel et al., 2017). Regarding SW, their formation is dependent upon cortico-cortical networks (Steriade et al., 1993; Timofeev et al., 2000; Shu et al., 2003). In concordance with their age-related global diminution, with dominant affection over the frontal and prefrontal areas (Mander et al., 2013; Dubé et al., 2015; Varga et al., 2016), the cortical volume of the medial prefrontal cortex, also the cortical thickness of the middle frontal gyrus, the superior parietal lobule, the superior temporal area were positively correlated with SW amplitude (Saletin et al., 2013; Dubé et al., 2015) activity (Varga et al., 2016) and density in both young and older adults (Dubé et al., 2015). All these cortical regions, such as the medial prefrontal cortex, also exhibit the most evident age-related cortical atrophy (Mander et al., 2013; Dubé et al., 2015). The age-related changes in SW characteristics and GM integrity are not independent of each other, as cortical thinning in these areas consistently and significantly attenuates the effect of age on SW density, amplitude, and activity (Mander et al., 2013; Dubé et al., 2015). These data indicate the age-related cortical atrophy in regions involved in the generation and propagation of sleep neuronal events as a mediator between age and age-related changes in sleep microstructure and quality.

Additionally, the integrity of WM and GM seems to moderate the effect of sleep on memory consolidation. The integrity of temporal WM tracts and hippocampal GM volume and its neural activity were predictive of SS impact on overnight motor memory consolidation (Fogel et al., 2017; Mander et al., 2017). This motor memory consolidation after sleep was significantly higher in young compared to older participants, even though the learning ability, i.e., the pattern of increasing performance during learning, did not differ between these groups (Fogel et al., 2017; Mander et al., 2017). In comparison to SS, slow-wave sleep strongly correlates with spatial (Varga et al., 2016) and declarative (Marshall et al., 2006; Backhaus et al., 2007; Mander et al., 2013) memory consolidation. Deficits in spatial and declarative overnight memory consolidation were proportional to the extent of SW activity impairment with age, and older adults again failed to gain the same benefits from sleep when compared to young counterparts (Mander et al., 2013; Varga et al., 2016). Moreover, medial prefrontal cortex volume significantly mediated the effect of age on the decrease in SW activity (Mander et al., 2013). The above stated implies that age-related decline in sleep-dependent memory consolidation may be dependent on WM and GM integrity.

## Discussion

The current review presents and attempts to synthetize the following conclusions:

(1) Brain atrophy and disruption of functional neuronal connectivity are hallmarks of brain aging. This conclusion is supported by a number of neuroimaging studies (Resnick et al., 2003; Sowell et al., 2003; Raz et al., 2004; Allen et al., 2005; Fjell et al., 2009; Mueller and Weiner, 2009; Eavani et al., 2018). However, the aging patterns are not unanimous, even in cognitively unaffected individuals (Eavani et al., 2018).
(2) Aging is associated with sleep shortening, fragmentation, and deterioration of its quality. Both, NREM and REM sleep are affected (Van Cauter et al., 2000; Ohayon et al., 2004). Even though the NREM deterioration seems to be more extensive (Van Cauter et al., 2000; Ohayon et al., 2004), NREM and REM are highly complementary, and in the long-run, equally important for brain homeostasis (Vyazovskiy and Delogu, 2014).
(3) Individuals with inadequate sleep length and/or quality are more commonly affected by brain atrophy and have lower cerebral functional connectivity. Short sleep duration and poor sleep quality are longitudinally associated with an increased cortical thinning rate (Sexton et al., 2014a; Spira et al., 2016; Tahmasian et al., 2020). During sleep, the brain is cleansed from toxic waste products (Xie et al., 2013), which might eventually trigger chronic inflammation, leading to subsequent tissue atrophy. Moreover, the glymphatic system—one of the major clearance mechanisms—operates predominantly during the sleep period (Demiral et al., 2019). Secondly, according to animal models, sleep also influences gene expression. Specifically, the expression of genes involved in phospholipid synthesis and oligodendrocyte proliferation significantly increases during sleep, sustaining the integrity of WM tracts (Bellesi et al., 2013). Furthermore, the expression of genes regulating the antioxidant system (Lungato et al., 2013), immune and stress responses, and neuronal plasticity (Cirelli et al., 2006) is sensitive to sleep deprivation. These data imply the role of sleep in brain trophic changes.
(4) Simultaneously lower GM and WM integrity is predictive of poorer sleep. Studies using mediation analysis models suggest that neuronal sleep oscillations, a proxy of the quality of sleep, are influenced by cortical integrity (Mander et al., 2013; Dubé et al., 2015; Liu et al., 2018). Specifically, when considering cortical thickness in the analysis, the significance of age in the prediction of sleep oscillatory events variance drops significantly (Mander et al., 2013; Dubé et al., 2015).

Taken together, sleep and brain structure and function are strongly interconnected. Brain structure impacts the interindividual variability of sleep oscillations, while sleep significantly affects brain metabolism, gene expression and, consequently, brain tissue integrity. Ergo, possible improvements in the duration and quality of sleep may represent a potent modifiable factor in the prevention of aging-related brain changes, ultimately also dementia and other neurodegenerative diseases. Considering the ubiquitous chronic sleep deprivation, we are in dire need of longitudinal multimodal studies, which could shed further light on the causality of the relationship between age-related alterations and sleep disturbances and possibly also on the extent and further qualities of the proposed bidirectionality.

In conclusion, the currently available literature implies a plausible and non-negligible interconnection between age-related alterations to the central nervous system integrity and sleep structure. Nevertheless, the true underlying causality of the relationship between sleep and aging remains elusive. Does poor sleep quality or quantity lead to accelerated brain atrophy, microstructural, and functional changes generally seen in aging? Or is the direction of the association inverse—is poor sleep quality only a secondary symptom of aging-related cerebral changes? Can we slow down or prevent some of the brain changes associated with aging by improving sleep quality and duration? Unfortunately, we do not know. Thankfully, the field of sleep research is going through a rapid evolution in available study methods, with much to be expected from novel polysomnographic approaches and imaging-derived metrics.

## Author Contributions

VK reviewed the literature and wrote the manuscript. DK, MB, and PF read and revised the manuscript. All authors approved the final manuscript.

## Conflict of Interest

The authors declare that the research was conducted in the absence of any commercial or financial relationships that could be construed as a potential conflict of interest.

## Publisher’s Note

All claims expressed in this article are solely those of the authors and do not necessarily represent those of their affiliated organizations, or those of the publisher, the editors and the reviewers. Any product that may be evaluated in this article, or claim that may be made by its manufacturer, is not guaranteed or endorsed by the publisher.
